# Preparation for airway management in Australia and New Zealand ICUs during the COVID -19 pandemic

**DOI:** 10.1371/journal.pone.0251523

**Published:** 2021-05-07

**Authors:** David J. Brewster, Christopher P. Nickson, Steve McGloughlin, David Pilcher, Vineet V. Sarode, Jonathan J. Gatward

**Affiliations:** 1 Intensive Care Unit, Cabrini Hospital, Malvern, Victoria, Australia; 2 Central Clinical School, Faculty of Medicine, Monash University, Melbourne, Victoria, Australia; 3 Intensive Care Unit, Alfred Health, Melbourne, Victoria, Australia; 4 Centre for Health Innovation, Alfred Health, Melbourne, Victoria, Australia; 5 School of Public Health and Preventive Medicine, Monash University, Melbourne, Victoria, Australia; 6 The Australian and New Zealand Intensive Care—Research Centre, Department of Epidemiology and Preventive Medicine, Monash University, Melbourne, Victoria, Australia; 7 Intensive Care Unit, Royal North Shore, Sydney, NSW, Australia; 8 The University of Sydney Northern Clinical School, Sydney, NSW, Australia; Azienda Ospedaliero Universitaria Careggi, ITALY

## Abstract

**Background:**

This paper aimed to describe the airway practices of intensive care units (ICUs) in Australia and New Zealand specific to patients presenting with COVID-19 and to inform whether consistent clinical practice was achieved. Specific clinical airway guidelines were endorsed in March 2020 by the Australian and New Zealand Intensive Care Society (ANZICS) and College of Intensive Care Medicine (CICM).

**Methods and findings:**

Prospective, structured questionnaire for all ICU directors in Australia and New Zealand was completed by 69 ICU directors after email invitation from ANZICS. The online questionnaire was accessible for three weeks during September 2020 and analysed by cloud-based software. Basic ICU demographics (private or public, metropolitan or rural) and location, purchasing, airway management practices, guideline uptake, checklist and cognitive aid use and staff training relevant to airway management during the COVID-19 pandemic were the main outcome measures. The 69 ICU directors reported significant simulation-based inter-professional airway training of staff (97%), and use of video laryngoscopy (94%), intubation checklists (94%), cognitive aids (83%) and PPE “spotters” (89%) during the airway management of patients with COVID-19. Tracheal intubation was almost always performed by a Specialist (97% of ICUs), who was more likely to be an intensivist than an anaesthetist (61% vs 36%). There was a more frequent adoption of specific airway guidelines for the management of COVID-19 patients in public ICUs (94% vs 71%) and reliance on specialist intensivists to perform intubations in private ICUs (92% vs 53%).

**Conclusion:**

There was a high uptake of a standardised approach to airway management in COVID-19 patients in ICUs in Australia and New Zealand, likely due to endorsement of national guidelines.

## Introduction

The COVID-19 pandemic has presented significant challenges to the international intensive care community during 2020. This included surge capacity planning, absenteeism due to staff furlough or infection, rapid staff training on the processes of donning and doffing personal protective equipment (PPE) and the introduction of novel clinical guidelines and practices. The airway management of COVID-19 patients has been identified as a significant risk to both staff (due to potential aerosol transmission) and severely hypoxic patients [1]. The Safe Airway Society of Australia and New Zealand published a consensus statement in March 2020 that was widely endorsed by specialty colleges and airway societies in Australia and New Zealand, including the Australian and New Zealand Intensive Care Society (ANZICS) and College of Intensive Care Medicine (CICM) [2]. These guidelines described airway management processes broadly consistent with those released less than two weeks later by the Difficult Airway Society in the UK [3]. Both of these guidelines make recommendations around team structure, clinical decision making, the intubating environment and staff training; as well as the use of cognitive aids, checklists, pre-packaged intubation trays and techniques such as videolaryngoscopy (VL).

There are 191 ICUs in Australia (119 public and 72 private). Private hospitals account for 33% of all current ICU beds. Both private and public ICUs were planned to surge in capacity to cope with the COVID-19 pandemic [4]. Some ICUs reported significant resource burden and urgent need for staff training in preparation for the COVID-19 pandemic [5]. By June 2020, 225 patients had been admitted to Australian intensive care units (ICUs) with COVID-19 disease and 58% of these patients required invasive mechanical ventilation. The median ICU length of stay for invasively ventilated patients was 16 days, ICU mortality was 15% and median peak COVID-19 ICU bed occupancy at this time was 14% [6]. These outcomes were noticeably different than the ICU mortality rates of 40–80% reported earlier in the pandemic abroad [7–10]. The reasons for this difference are unclear, but it seems likely that hospitals in Australia and New Zealand benefitted from extra time to prepare their response for the COVID-19 pandemic through the writing and endorsement of clinical guidelines and the training of staff.

We aimed to provide a detailed snapshot of the airway practices and staff training within the ICUs in Australia and New Zealand specific to the management of patients presenting with COVID-19 during the pandemic. Emphasis was placed on the use of airway techniques and equipment, staff training and the uptake of endorsed guidelines, cognitive aids and checklists. This study also aims to inform whether consistent airway management practice within ICUs was seen during the pandemic in Australia and New Zealand.

## Methods

### Ethical consideration

The study received ethics approval from the Cabrini Institute Ethics Committee (06-15-06-20). Participation in the study was voluntary and completion of the questionnaire implied consent.

### Study design, participant population and survey instrument

A web-based clinical preparation and practice survey specific to ICUs in Australia and New Zealand was designed to determine their routine airway management practices during 2020 specific to adult patients with confirmed or suspected COVID-19. All authors designed and revised the questionnaire before distribution. The questions were aimed to help obtain data to describe institutional preparation and airway management within the ICU during the COVID-19 pandemic. Existing airway guidelines were used to aid with question development. All two hundred ICU directors in Australia and New Zealand were emailed a questionnaire by ANZICS in September, 2020. The email contained a link created by the cloud-based software Qualtrics (UT, USA). This software collected and reported all data from the responses. The questionnaire was left open for a period of three weeks. S1 Appendix is a summary of the survey questions. All ICU directors were sent a second reminder email about the questionnaire.

### Statistical analysis

Questionnaire data were analysed to determine basic ICU demographics (private or public, metropolitan or rural) and location as well as equipment purchasing, airway management practices, guideline uptake, checklist and cognitive aid use and staff training relevant to airway management during the COVID-19 pandemic.

All responses were tabled for comparison. Data are presented as both total responses and percentages of responses. Cross-tabulations were used to analysed the data and to establish whether there was any significant effect of unit demographics or location on airway procedures, use of airway equipment and support for airway training. The software determined the overall significance using a Chi-squared test.

## Results

**Fig 1:**
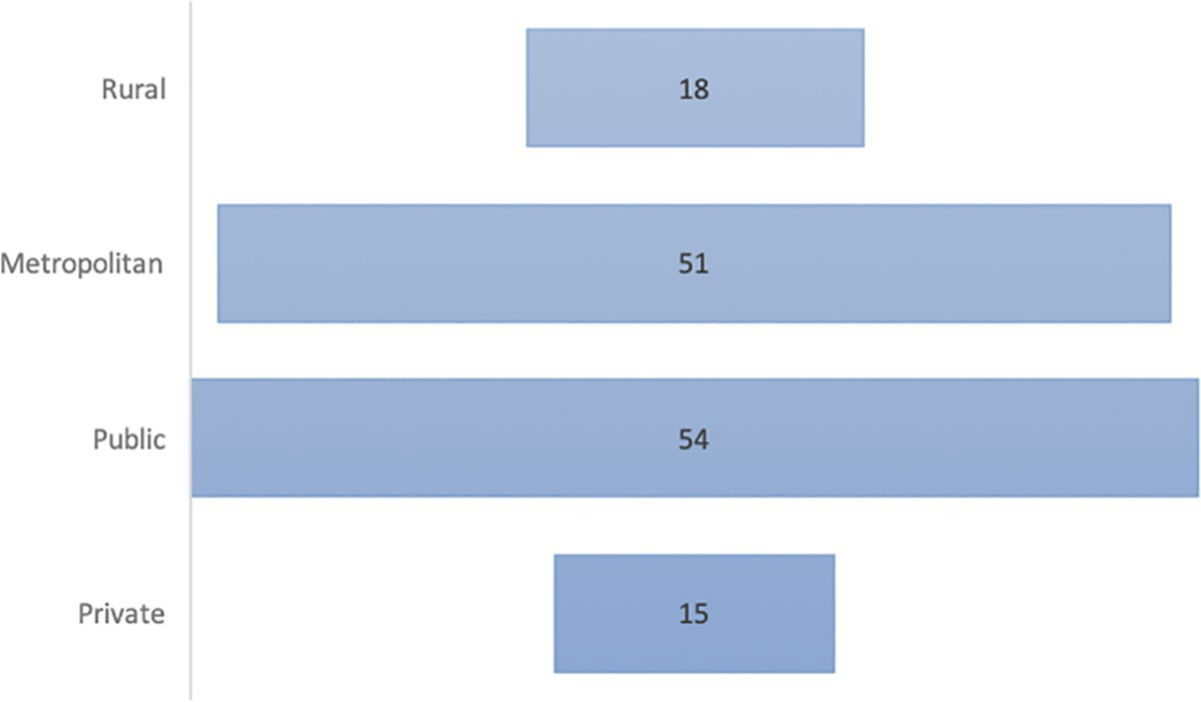
Demographics of ICU responders.

**Fig 2:**
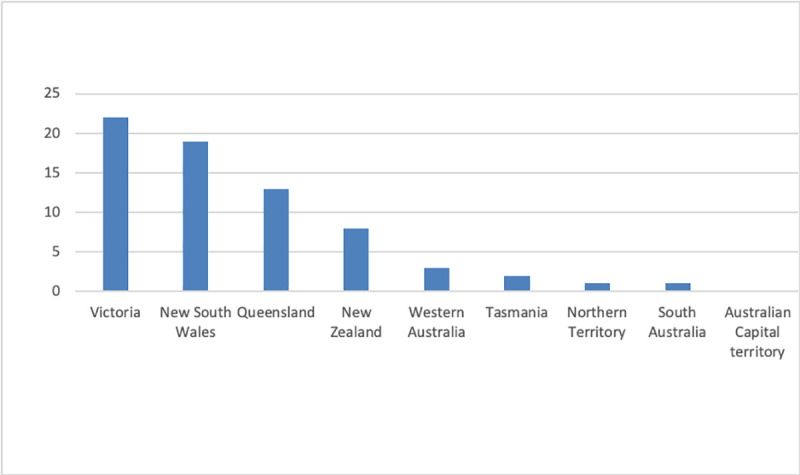
Locations of ICU responders.

69 ICU directors responded (63 surveys completed in full, 6 partially completed). Data are presented as both total counts and percentages of completed responses to individual questions. The demographics and locations of the responding units are shown in Figs 1 and 2.

The preparation of intubation environments in the ICU and new equipment purchased is shown in Table 1. The introduction of training specific to airway management of patients with COVID-19 was reported by 66/67 ICUs (98.5%). Details of this training are shown in Table 2. The reported use of specific intubation guidelines, checklists and specific equipment is presented in Table 3.

**Table 1:** New equipment purchased for COVID-19 airway management in ICU.

	**n**	**%**
***Intubation environment for COVID-19 ICU patients*** *		
An existing negative pressure room in ICU	52	75.4
A newly developed negative pressure room	11	21.2
A standard ICU room	11	21.2
***New airway equipment purchased for COVID-19 pandemic*?**		
Yes	53	77.9
No	15	22.1
***Airway equipment purchased for COVID-19 pandemic***		
VL monitor(s)	39	57.4
Disposable VL blades	31	45.6
HMEs/viral filters	33	48.5
Ventilators	40	58.8
Other airway equipment	7	10.3
**New personal protective equipment (PPE) purchased for staff involved in the airway management of COVID-19?**		
Yes	56	82.4
No	12	17.6
**Pre-packed COVID-19 intubation equipment trays prepared?**		
Yes	50	73.5
No	18	26.5

*(multiple answers possible as two different intubation rooms in one ICU possible).

**Table 2:** New training for airway management of COVID-19 in ICU.

	**n**	**%**
***Type of training for the airway management of COVID-19 patients in ICU***		
Inter-professional	66	98.5
Simulation based (e.g. intubation of a manikin in airborne PPE)?	65	97
Include use of cognitive aids	59	88
Include PPE donning/doffing	66	98.5
Include training of PPE “spotters”	63	94
***Shared training program/resources for the airway management of COVID-19 patients between ICU and other departments (e*.*g*. *ED or anaesthesia)*?**		
Yes	58	86.6
No	9	13.4

*(multiple answers possible as two different intubation rooms in one ICU possible).

**Table 3:** New guidelines, equipment and processes for airway management of COVID-19 in ICU.

	n	%
***Did your unit adopt airway management guidelines to be used for the intubation of COVID-19 patients*?**		
Yes	59	89.4
No	7	10.6
***New airway guidelines adopted***		
Safe Airway Society	32	54.2
Difficult Airway Society, UK	12	22.2
Hospital specific	11	18.6
Other	4	6.8
***Cognitive aids for intubation of COVID-19 patients on ICU airway trolleys*?**		
Yes	55	83.3
No	11	16.7
***Cognitive aids chosen***		
Hospital Specific	34	61.8
Safe Airway Society	19	34.5
Other	2	3.6
***Has a patient with suspected or confirmed COVID-19 been intubated in your ICU*?**		
Yes	52	
No	14	
***Most likely “intubating clinician” for COVID-19 patient in ICU*?**		
Consultant intensivist	39	60.9
Consultant anaesthetist	23	35.9
Trainee	2	3.1
***Total ICU staff in room for intubation***		
2	4	6.2
3	38	59.4
4	21	32.8
>4	1	1.6
***Consultant (specialist) staff in room for intubation***		
0	0	0
1	37	57.8
2	24	37.5
>2	3	4.7
***Equipment/Personnel/Processes used for all intubations of COVID-19 patients in ICU*?**		
Video-laryngoscopy	59	93.7
PPE “spotters”	56	88.9
Team Huddle	59	93.7
Checklists	59	93.7
Face shields	60	95.2
N95 masks	58	92.1
PAPR	8	12.7
Methods of pre-oxygenation		
Face mask (two hand grip)	50	76.9
Non-Rebreather Mask	19	29.2
High Flow Nasal O_2_	14	21.5
Non-Invasive Ventilation	10	15.4
Dedicated Intubation Teams		
Yes	41	61.2
No	26	38.8

*(multiple answers possible as two different intubation rooms in one ICU possible).

Cross tabulation of all data was done to look for any significant differences in responses between ICUs from New Zealand and different Australian states and territories, as well as both public and private and rural and metropolitan ICUs. Significant differences are reported shown Table 4.

**Table 4:** Statistically significant results of data cross tabulation.

	Public	Private	*p* value
***New airway processes***			
New airway guidelines adopted	49 (94%)	10 (71%)	*0*.*01*
***Intubating clinician***			
Intensivist	27 (52.9%)	12 (92.3%)	0.04
Anaesthetist	22 (43.1%)	1 (7.7%)	0.04
Trainee	2 (3.9%)	0	*0*.*03*

## Discussion

This study provides a snapshot of the airway practices and airway specific training in ICUs in Australia and New Zealand during the COVID-19 pandemic. Almost universal simulation-based inter-professional airway training (97%) was reported, along with the use of VL (94%), intubation checklists (94%), cognitive aids (83%) and PPE “spotters” (89%) during the airway management of patients with COVID-19. Although some hospitals still wrote their own airway management protocols, the majority adopted the endorsed SAS or guidelines (or similar DAS guidelines). Tracheal intubation was almost always performed by a Specialist (97% of ICUs), who was more likely to be an intensivist than an anaesthetist (61% vs 36%). The significant differences shown between public and private ICUs were the more frequent adoption of specific airway guidelines for the management of COVID-19 patients in public ICUs (94% vs 71%) and the reliance on specialist intensivists to perform intubations in private ICUs (92% vs 53%). The significant burden of purchasing and preparing new equipment for the airway management of COVID-19 patients in ICU is also reported, with the majority of ICUs needing to purchase new VL equipment, ventilators and PPE.

The differences between public and private ICUs may be explained by the absence of large anaesthetic departments (with trainees) within private hospitals to support both airway guideline uptake and airway management within the ICU. It should be noted, this may not be true of all private hospitals, as some large private hospitals may now have similar training and structures to their public partners.

Brewster et al. described the airway practices in tertiary ICUs in Australia and New Zealand in 2018, reporting that most intensivists had a modest volume of tracheal intubation practice, supervised intubations by trainees in ICU more commonly than performed them, and 43% of intensivists reported utilizing VL on first attempt in ICU [11] They also demonstrated a variable uptake of multiple different guidelines for the management of the airway crisis of “can’t intubate can’t oxygenate” (CICO), with no specific CICO guideline preferred by more than 28% of specialists. These results were supported by a second paper by Toolis et al. following a survey of intensivists in Australia and New Zealand in 2019 [12] They also reported modest annual intubations amongst intensivists, as well as a 43% usage of VL during intubations in ICU [13].

Our data suggest a dramatic increase in the use of VL in ICU (up from 43% in 2018 to 94% in this pandemic). This may be due to the guideline recommendation of VL in this specific patient group, but might also reflect a trend towards the adoption of VL for other critically ill patients, which has previously been limited by cost and the availability of equipment. It remains to be seen whether the increased use of VL that we have observed will persist after the current pandemic.

The universal use of inter-professional simulation-based airway training during the COVID-19 pandemic by staff in Australian and New Zealand ICUs is heartening given the known benefits of team-training in ICU [13]. It also addresses the desire expressed by CICM fellows for more airway CPD [11, 12]. The addition of mandatory airway training to the CICM CPD program would likely benefit patient safety and maintain intensivist skills even beyond the pandemic. Simulation has advantages beyond staff training that have also been used during the COVID-19 pandemic, such as the testing of systems and cognitive aids [14, 15]. Simulation allows generic guidelines to be rapidly modified to local contexts and ergonomics issues to be addressed through changes to training, equipment, and environment [16]. Simulation allowed ICU staff to familiarise themselves to the new intubation environment caused by COIVD-19 and the use of PPE. Communication was more difficult in PPE, and the new recommended processes within intubation guidelines to protect staff from infection required practice.

The widespread adoption of guidelines on the airway management of COVID-19 patients is reassuring. Standardised practice and training have numerous benefits and aims to not only provide a gold standard of care but also reduce human error [17, 18]. Rapid endorsement by numerous specialty colleges and airway societies likely increased the distribution, awareness and uptake of these guidelines as ICUs prepared for the pandemic. The inclusion of cognitive aids and checklists in these guidelines likely contributed to our finding that they are being used widely during the pandemic. The fact that these were mostly hospital-specific (62%) is consistent with recommendations that cognitive aids should be adapted to local context, and also undergo simulation-based usability testing [19]. A well designed generic cognitive aid, provides a useful starting point for local adaptation and implementation. This practice was also seen in Europe, with the use of checklists and cognitive aids encouraged after the early experiences with COVID-19 in Italy [20, 21].

This study has some limitations. All surveys have bias relating to response rates. 69 responders are a large sample of ICUs in Australia and New Zealand, but still represent only 35% of all ICUs surveyed. We suggest that units without any experience with COVID-19 likely did not respond. There may also be recall bias due to the recollections of ICU directors, as opposed to the individual clinicians. However, the directors hopefully give an accurate reporting of the processes and equipment purchasing that were in place at the individual ICUs. The quality of the airway practices reported is not known. The number of respondents was slightly over-represented in Victoria and under-represented in South Australia and Western Australia, but this may make the survey more representative, given the significantly increased volume of COVID-19 patients treated in ICU in Victoria compared to the rest of Australia and New Zealand at the time of the survey. Future research into the airway practices of ICUs in Australia and New Zealand may choose to focus on the ongoing use of VL, simulation training, checklists and cognitive aids and any substantial improvement in both patient or staff outcomes.

In summary, our survey indicates that there was a high uptake of a standardised approach to airway management in COVID-19 patients in ICUs in Australia and New Zealand. This approach included the use of endorsed guidelines, checklists, cognitive aids and "PPE Spotters”. There was a large increase in the use of VL. There was almost universal use of interprofessional simulation to train staff, optimise ergonomics and embed new processes and equipment.

We believe that this type of standardised approach, accompanied by checklists, cognitive aids and simulation-based training, as well as dedicated rooms for intubation within the ICU, could be applied to all ICU airway management in Australia and New Zealand beyond the pandemic and into the future.

## Supporting information

S1 Appendix Summary of survey questions. (DOCX)
