# Supplementary material for: Preparation for airway management in Australia and New Zealand ICUs during the COVID -19 pandemic
Source: PLoS One. 2021 May 7;16(5):e0251523. doi: 10.1371/journal.pone.0251523 (PMC8104394; doi:10.1371/journal.pone.0251523)
Supplement: S1 Appendix — (DOCX) [file pone.0251523.s001.docx]

S1 Appendix - Summary of survey questions

Unit demographics

Preparation of ICU for COVID-19

- Intubation room
- Equipment for COVID-19 airway management
- Personal protective equipment (PPE) for staff involved in the airway management of COVID-19
- Pre-packed COVID-19 intubation trays

Preparation of staff for COVID-19 airway management

- Use of “intubations teams”
- Leader of intubations in ICU
- Training of staff for the airway management or intubation of COVID-19 patients
- Type of training (eg. simulation, inter-professional) for the airway management or intubation of COVID-19 patients
- Use of cognitive aids for intubation of COVID-19 patients
- Training of ICU staff for PPE donning and doffing for intubation
- Training of staff as “spotters” for PPE donning and doffing for intubation
- Shared education programs or shared education resources for the airway management of COVID-19 patients between your ICU and other departments (e.g. ED or anaesthesia)

Intubation process for COVID-19 patients

- Adoption of airway management guidelines to be used for the intubation of COVID-19 patients
- Specific airway guidelines adopted for the intubation of COVID-19 patients
- Hospital specific protocol for the intubation of COVID-19 patients
- Cognitive aids for intubation of COVID-19 patients available on airway trolleys
- Intubating clinician of COVID-19 patients in the ICU (anaesthetist, intensivist, trainee, other)
- Number of staff were typically in the room during intubation
- Number of consultant staff (ICU, anaesthetic or other) in the room during intubation
- Use of a designated “spotter” for PPE donning/doffing
- Use videolaryngoscopy by Intubating clinician of COVID-19 patients in the ICU
- Use of apneic oxygenation technique and other methods of pre-oxygenation used prior to intubation
- Use of a team huddle prior to the intubation process
- Use of an intubation checklist and cognitive aids for intubation of COVID-19 patients in the ICU
- PPE for intubation of COVID-19 patients in ICU
